# Why vitamin D for cancer patients?

**DOI:** 10.3332/ecancer.2009.160

**Published:** 2009-09-30

**Authors:** S Gandini, FP Francesco, H Johanson, B Bonanni, A Testori

**Affiliations:** 1 Epidemiology and Biostatistics Division; 2 Melanoma and Muscle-Cutaneous Sarcomas Division; 3 Cancer Prevention and Genetics Division, European Institute of Oncology, Milan, Italy

## Abstract

Several epidemiological, pre-clinical and clinical studies support Vitamin D as a preventive and therapeutic cancer agent.

**Background:**

**Vitamin D and cancer:** calcitriol, the biologically active form of vitamin D (1,25(OH)D), exerts its effects mainly through binding to nuclear vitamin D receptor (VDR). Calcitriol has been shown to be an anti-proliferative, pro-differentiation, pro-apoptotic agent and an inhibitor of cell migration. Animal and human *in vitro* studies strongly indicate that vitamin D may have benefits for many forms of cancer. Inadequate levels of circulating 25-hydroxy-vitamin D (25(OH)D) are associated with an increased risk and poor prognosis of several types of cancer.

**Vitamin D and melanoma:** cutaneous malignant melanoma (CMM) represents a major public health issue: rates in Italy have almost doubled in the last decade and CMM is frequent among young adults. For resected stage II melanoma no standard adjuvant treatment exists and five-year overall survival is about 70%.

Cultured melanoma cells can synthesize calcitriol from 25(OH)D and express the VDR. Moreover, 1,25(OH)D has anti-proliferative and pro-differentiation effects in human melanoma cells. 1,25(OH)D has been shown to induce apoptosis in human melanoma cell lines and has an inhibitory effect on the spreading of melanoma cells *in vitro*.

**Preliminary results on vitamin D:** epidemiological data indicate that vitamin D deficiency is relatively common in Europe. In an Italian study, we found that 85% of the participants had insufficient levels of 25(OH)D. We have shown through a meta-analysis of randomized trials that vitamin D supplementation is associated with a significant reduction (7%) in total mortality in healthy subjects and an association between VDR and 25(OH)D and CMM progression has also been demonstrated. We have also reported significant associations between VDR polymorphisms and incidence of skin cancer. In early supplementation trials, the lack of effect on cancer incidence has been attributed to insufficient vitamin D supplementation, stressing the need to better study vitamin D bioavailability.

In fact, a recent IARC report highlighted the need for new randomized trials, based on results from our meta-analyses on 25(OH)D serum levels and cancer risk.

**Clinical trial and biomarkers studies:** in order to assess whether vitamin D supplementation could improve prognosis of CMM, an Italian multi-centre trial in stage II resected melanoma patients was planned to monitor changes in 25(OH)D. The study will address two important questions regarding the relationship between the biology of VDR and (1) vitamin D metabolism, and (2) CMM prognosis. This will involve investigating the association between VDR polymorphisms and Breslow thickness, the most important prognostic factor of CMM, and between 25(OH)D serum level, vitamin D supplementation and VDR.

We will also evaluate at baseline whether VDR polymorphisms are associated with Breslow thickness and whether we obtain significant increase in 25(OH)D serum levels during the first year of supplementation. We will quantify the percentages of patients who have desirable levels of 25(OH)D and, if they don’t, the mean time to reach that level. The findings from this study will be of great interest because vitamin D could have anti-cancer benefits for a wide spectrum of cancers.

## Background and rationale

### Biological background

Cancer chemoprevention uses natural, synthetic or biologic agents to reverse, suppress or prevent carcinogenic progression. Genetic changes exist throughout the process and increase the likelihood that one or more pre-malignant and malignant lesions may develop within that process. Multi-step carcinogenesis describes a stepwise accumulation of alterations, both genotypic and phenotypic. Arresting one or several of the steps may impede or at least delay the development of cancer.

Several epidemiological, pre-clinical and clinical studies support vitamin D as a preventive and therapeutic cancer agent, for a wide spectrum of cancers.

Calcitriol (1,25-dihydroxyvitamin D [1,25(OH) D]), the hormonal derivative of vitamin D, has been established since the 1980s as an anti-proliferative and pro-differentiation agent and as a pro-apoptotic agent and an inhibitor of cell migration, which may imply an inhibitory effect on cancer [1].

Vitamin D is indeed more like a hormone and not strictly a vitamin according to the classical criteria that an essential nutrient is a substance the body cannot synthesize in sufficient quantities itself. Also, vitamins are usually involved in biochemical reactions, while 1α,25-dihydroxyvitamin D exerts its action via VDR.

Vitamin D represents a group of fat-soluble pro-hormones, the two major forms of which are vitamin D_2_ (or ergocalciferol) and vitamin D_3_ (or cholecalciferol). Endogenous synthesis of vitamin D_3_ takes place in the skin under the influence of ultra-violet B (UVB) radiation. Exogenous vitamin D_2_ or D_3_ comes from dietary intake. The overall vitamin D intake is the sum of cutaneous vitamin D and nutritional vitamin D.

Vitamin D on its own has no physiological action. To be physiologically active, vitamin D must first be hydroxylated in the liver by the enzyme 25-hydroxylase, encoded by CYP27A1 (also called the 25-hydroxylase or 25(OH)D), into 25-hydroxyvitamin D. The 25-hydroxyvitamin D is inactive, and an additional hydroxylation in the kidney by the enzyme 1α-hydroxylase, encoded by CYP27B1 (also called 41α-hydroxylase), is necessary to produce the physiologically active vitamin D metabolite, calcitriol or 1,25(OH)D. When 1,25(OH)D is sufficiently available, the enzyme mitochondrial protein encoded by CYP24A1 metabolises the 1,25(OH)D into 1α,24,25-dihydroxyvitamin D, which is further catabolised to calcitroic acid. 25(OH)D and 1,25(OH)D are transported in serum by the vitamin D-binding protein (GC). Ahn *et al* systematically investigated the association of 48 SNPS in four vitamin D metabolising genes (CYP27A1, GC, CYP27B1 and CYP24A1) with serum 25(OH)D levels. Four tagSNPS in GC, the major serum 25(OH)D carrier, were associated with 25(OH)D levels [2].

### VDR and cancer risk

The vitamin D receptor (VDR) gene is involved in multiple pathways that may be important in the aetiology of cancer. The importance of dietary calcium, vitamin D, energy and fat in modifying the association between VDR genotype and cancer risk has been shown repeatedly. In addition to these dietary factors that modify cancer risk, BMI and age also appear to be ‘effect modifiers’ of the association between VDR and cancer.

We performed a meta-analysis on the association between the two most studied VDR polymorphisms (FokI and BsmI) and cancer at any site. When comparing FokI ff with FF carriers, we found a significant increase in skin cancer (SOR; 95% confidence intervals (CI): 1.30; 1.04–1.61) and breast cancer (SOR; 95%CI: 1.14; 1.03–1.27) risk. For the same genotype comparison, we found a significantly higher risk of cancer when we pooled estimates from cancer sites possibly associated with vitamin D levels (prostate, breast, skin, ovary, non-Hodgkin lymphoma). A significant reduction in prostate cancer risk was observed for carriers of BsmI Bb compared with bb genotype (SOR; 95%CI: 0.83; 0.69–0.99). In Caucasian populations, both Bb and BB carriers had a significant reduced risk of cancer at any site. In conclusion, this meta-analysis showed that VDR FokI and BsmI polymorphisms may modulate the risk of cancer of the breast, skin and prostate and possibly affect cancer risk at any site in Caucasians [3].

### Sources of vitamin D

There is good evidence from randomized trials that a dietary intake of vitamin D increases serum levels of 25-hydroxyvitamin D [4]. However, only a few foods naturally contain appreciable enough amounts of vitamin D to have an impact either through the form of cholecalciferol (vitamin D3) derived from animal sources, or ergocalciferol (vitamin D2), from plant food. It has been verified that fatty fish such as salmon, mackerel and bluefish, cod-liver oil, egg yolks, sun-dried mushrooms and yeasts are excellent sources of vitamin D_3_.

Most of an individuals vitamin D supply is provided through endogenous synthesis of vitamin D_3_ upon sunshine exposure and will depend on amounts of UVB reaching earth surface, on skin surface exposed to UVB and on skin pigmentation. Many factors influence the amount of UVB radiation reaching the Earth’s surface.

In summary, these factors include:

- UVB is mainly present in sunlight between 10 am and 3 pm solar hours.
- The season: UVB is more abundant during the summer or the hot season.
- Place of residence.
- Meteorology: clouds may filter out much of the UVB, even at the equator.
- The UVB-induced synthesis of vitamin D_3_ in human skin is additionally dependent on several factors, including:
  time spent out-of-doors (evaluation of continuous pattern versus intermittent pattern of sun exposure);
  skin type, pigmentation;
  age of subjects, as endogenous skin synthesis capacity decreases with age;
- Sun protection habits, mainly in populations where clothing (garments and veils) covers most of the skin surface.

Results from a study by Adams *et al* [61] also show that the serum level of 25(OH)D is more stable than vitamin D. Because of its relatively long half-life (τ1/2 = 12.9 (SD: 3.6 d)) [5], the serum 25(OH)D level is considered as the best gauge of individual vitamin D status.

### Vitamin D and mortality from all causes

We published a meta-analysis of published randomized trials, which showed a significant reduction of 7% in total mortality (RR 0.93, p<0.05) in subjects taking vitamin D. Eighty-two percent of patients received vitamin D_3_ (cholecalciferol), the remaining vitamin D_2_ (ergocalciferol), either orally or by injection. Average daily doses ranged from 300 to 2000 IU. Treatment ranged from daily to four-monthly, and follow-up ranged from six months to seven years. Our main recommendation in light of the results from this study was the conduction of large population-based randomized trials of prolonged vitamin D_3_ treatment [6]. The Netherlands Longitudinal Aging Study examined, during a six-year follow-up, the risk of death of 1260 community dwelling people 65 years old or more according to serum 25(OH)D levels measured at baseline [7]. The results indicated that subjects with serum 25(OH)D levels lower than 20 ng/ml had a mortality risk associated with steadily lower levels (log-rank test: p<0.0001).

In the Third National Health and Nutrition Examination Survey (NHANES III, USA) 13,331 adults, 20 years or older, were followed for a median of 8.7 years [8]. There were 1806 deaths, 777 from cardiovascular disease (CVD) and 424 from cancer. Serum 25(OH)D levels below 17.8 ng/ml were associated with a 26% increased rate of all-cause mortality [mortality rate ratio (95% CI: +8%; +46%)].

### Epidemiological evidence and optimal serum levels of 25(OH)D

So far epidemiological data for cancer prognosis argue for an overall positive role of sun-induced vitamin D. The ratios of death rates to incidence rates suggest that cancer prognosis improves with decreasing latitude. UV exposure is a realistic explanation since it is unlikely that cancer treatment is better in Australia than in the United Kingdom [9].

Genetic variations would be phenotypically apparent as inter-individual variations in limiting rates of vitamin D synthesis in the skin, hydroxylation in the liver and in the kidney, transport, metabolism, degradation would ultimately influence individual vitamin D status. These genetic differences would also be reflected in the numerous variants of the VDR, some of which have different affinity for 1,25(OH)D.

In general modern society is vitamin D deprived compared with prehistoric humans. The concentrations of 25(OH)D observed today are based on contemporary cultural norms (clothing, sun avoidance, food choices and legislation).

Inadequate levels of circulating 25(OH)D are associated with an increased risk and poor prognosis of several types of cancer [10–12].

Giovannucci estimated the relationship between plasma 25(OH)D and several risk factors (skin colour, obesity, latitude and physical activity) for cancer. The associations between these factors and total cancer incidence and mortality in their large cohort (∼50,000 subjects) could be accounted for an by an increment of 25 nmol/l in 25(OH)D, which reduces mortality by 29% for all cancers and 45% for digestive cancers [10]. These results are consistent with a marked direct effect of vitamin D on cancer incidence and mortality, but they could be at least partly due to confounding factors. This can be resolved only by a randomized trial.

A prospective study examined the association between pre-diagnostic 25(OH)D levels and mortality in patients diagnosed with colorectal cancer within the Nurses’ Health Study (NHS) and the Health Professionals Follow-Up Study (HPFS). This study showed that higher pre-diagnostic plasma 25(OH)D levels were associated with a significant improvement in overall survival [13]: individuals with vitamin D levels in the highest quartile were 48% less likely to die (from any cause) than those with the lowest vitamin D levels (adjusted HR of 0.52: 95% CI, 0.29 to 0.94), with a linear and statistically significant trend. The odds of dying from colon cancer specifically were 39% lower (HR of 0.61; 95% CI, 0.31 to 1.19). The authors highlight the need for well-designed prognostic studies among cohorts of cancer patients, in which exposure assessments are uniformly obtained after diagnosis and information on prognostic tumour characteristics is also available. Several studies show promising results, but a randomized trial among cancer patients would be essential to obtain an unequivocal answer on whether cancer patients should be advised to take vitamin D supplements [14]. A German cohort study of 3299 patients referred for coronary angiography recorded, during a median 7.8 years of follow-up, 736 deaths, among which 95 were attributed to a cancer [15]. After adjusting for common confounders (e.g. sex, age, obesity, smoking status, physical exercise) the study found a twofold increased risk (95% CI: 1.01–3.8) for a cancer-related death in patients with serum 25(OH)D levels below 15 ng/ml, as compared to patients with higher serum concentrations. This risk did not vary in patients with serum levels higher than 15 ng/ml.

A working group of experts, organized by the International Agency for Cancer Research, carried out meta-analyses on 25(OH)D serum levels and colorectal cancer incidence. We observed a significant reduction in risk comparing the highest levels versus the lowest level of 25(OH)D; pooled RR: 0.6 (95%CI: 0.4, 0.7), with a significant dose-response effect. Among the studies included, the lowest values of 25(OH)D for the upper categories in average were 34 mg/ml and the upper levels of the lowest category was 18 mg/ml [59].

Vitamin D deficiency is defined by most experts as a 25(OH)D level of less than 20 ng/ml (50 nmol/l) [16,17]. By these standards both the European and US populations are vitamin D insufficient or deficient. The total 25(OH)D, that is 25(OH)D_2_ plus 25(OH)D_3_, is what physicians need to be aware of in their patients.

The estimated optimal serum 25(OH)D level for prevention of colorectal carcinoma and adenoma is 90 nmol/l or higher, a conclusion supported by the NIH-sponsored symposium on vitamin D and cancer [18,19].

In a trial (IEO 007) carried out by our Cancer Prevention and Genetics Division in pre-menopausal Italian women at high risk of breast cancer, we found that 85% had at insufficient level of 25(OH)D, considering as cut-off point 75nmol/l. The results were presented at the AACR (2008).

Current efforts to assess optimal serum concentrations of 25(OH)D generally focus on bone health in older white persons, and the common definition of the optimal level has been the concentration that maximally suppresses serum parathyroid hormone (PTH). In most persons, the optimal level cannot be reached with the currently recommended intake of 200 and 600 IU/d for younger and older adults, respectively.

The Women’s Health Initiative (WHI) in the USA [20], a randomized trial evaluating the influence of 400 IU of vitamin D supplementation per day, plus calcium, showed negative results on all cancer risk. However, findings from the nested case-control study demonstrated a significant inverse trend with lower baseline levels of serum 25(OH)D associated with an increased risk of colorectal cancer (p for trend = 0.02). Commentaries on the negative findings of the WHI trial suggest that too-low vitamin D doses, too-short trial duration, addition of calcium and low compliance to supplementation may be an explanation [21,22].

The results of the nested case-control study organised within the WHI trial including colorectal cancer cases and 317 matched controls, showed that the risk of colorectal cancer increased with decreasing serum 25-hydroxyvitamin D levels in subjects with low-vitamin D status at baseline, before use of supplements or placebo. Results from these trials stress the need to better study vitamin D bio-availability.

A recent study on prognostic effects of circulating 25(OH)D in a cohort of patients with early breast cancer found that deficient levels of vitamin D were associated with higher grade tumours, suggesting that the prognostic effect of vitamin D may be due, in part, to the development of higher grade tumours in vitamin D-deficient women, consistent with a potential role of vitamin D in breast carcinogenesis [23].

### Safety and toxicity of vitamin D

Cholecalciferol accumulates in adipose and muscular tissue to be available when the body needs it.

A daily treatment of 2000 IU of Vitamin D_3_, the safe upper-intake limit as defined by the National Academy of Sciences, should increase circulating 25(OH)D to the desired level.

Thus 2000 IU is the safe recommended daily allowance even at the higher end of the normal 25(OH)D serum concentration level [24–29].

All the published reports of Vitamin D toxicity with convincing evidence of hypercalcemia involve serum 25(OH)D concentrations well above 200 nmol/l, which requires a daily intake of more than 40, 000IU, and which could be conservatively considered the lowest observed adverse effect level (LOAL).

Vitamin D intoxication is observed when serum levels of 25-hydroxyvitamin D are greater than 150 ng/ml (374 nmol/l). Vitamin D intoxication is extremely rare but can be caused by inadvertent or intentional ingestion of excessively high doses. Doses of more than 50,000 IU/d raise levels of 25(OH)D to more than 150 ng/ml (374 nmol/l) and are associated with hypercalcemia and hyperphosphatemia [30,31,32].

Trivedi *et al* in their trial on fractures and mortality reported no side effects in over 2000 subjects aged 65 or over receiving 2.5 mg po four-monthly or placebo for five years [33]. The Women’s Health Initiative trial reported a 17% increase in the risk of kidney stones among post-menopausal women receiving 10 μg of vitamin and 1000 mg of elemental calcium per day [34], but this was probably due to the calcium supplements, and not the vitamin D supplement. Other randomized trials testing vitamin D supplementation did not report higher incidence of kidney stones [4].

### Compliance

In the study by Trivedi *et al* a compliance of at least 80% (≥ 12 of 15 tablets posted four-monthly over five years) was achieved in 76% of randomized subjects. In this study, subjects received tablets four-monthly, with a freepost reply to report whether the tablet was taken and reasons for not taking it [33]. However, in the WHI study compliance to supplementation was low. Throughout the entire trial duration, only 50–60% of women took 80% of the scheduled supplementation regimen [20].

In our meta-analysis of 18 randomized trials on intakes of vitamin D and overall mortality, eight of these trials reported increases in serum 25-hydroxyvitamin D level in the intervention group, but variations ranged from a factor 1.4–5.2 mainly because of compliance issues [6].

### Vitamin D and melanoma, epidemiological evidence

Relatively few epidemiologic studies have directly addressed the relationship between vitamin D and the incidence or prognosis of cutaneous malignant melanoma (CMM).

Increasing incidence of skin cancer starting around the 1950s has been described in all light-skinned populations. These increases concerned all types of skin cancer. The most recent cancer registry data show that skin cancer incidence is still rising in nearly all light-skinned populations. Cutaneous malignant melanoma represents a major public health problem in many countries throughout the world. Over the past several decades, its incidence has increased more rapidly than that of any other cancer, although fortunately this increase has not been accompanied by a similar rise in mortality rates, possibly reflecting beneficial effects of awareness and prevention programmes. In Italy, the age-adjusted incidence is 4.6 per 100,000 person per year in males and 5.5 in females, with higher mortality rates being documented in Northern rather than in Southern Italy [35–37]. In Italy, the incidence of CMM has increased: rates have almost doubled in the last ten years. CMM is a disease of young subjects (>50% of all cases are diagnosed below the age of 60 years), and for Stage II melanoma no treatment can be offered to the patients after surgery.

The CMM fatality over five years was observed to be 40–60% less in people with a history of high sun exposure independently of body site, thickness, mitotic rate and early detection behaviour. The study by Berwick *et al* involved mostly patients with early stage melanoma, and it is plausible that the known anti-proliferative and anti-angiogenic properties of vitamin D may be inversely associated with melanoma progression. However, it should be noted that solar elastosis and other sun exposure markers in the study reflected pre-diagnostic exposures to sunlight [38]. Sunburn and intermittent sun exposure are well-known risk factors for CMM; however, continuous pattern of sun exposure seems to be inversely associated to melanoma [39,40]. Thus, it is not clear whether the results of Berwick *et al* reflect benefits related to continued sunlight exposure and an association with vitamin D intake, or a different pathogenesis in melanomas that arise in persons at risk to develop actinic skin damage.

Exposure to UVB increases endogenous vitamin D synthesis and risk of skin cancer. However, skin synthesis of vitamin D is self-limited and in light-skinned people fades away after 5–10 minutes. Longer durations of sun exposure will not further increase vitamin D, but will increase skin cancer risk. Furthermore, within an IARC working group, we showed that exposure to artificial UV light from sun beds increases the risk of melanoma and SCC, especially when the first exposure takes place before 35 years of age [41].

The incidence of melanoma of the skin on intermittently exposed sites is reduced among outdoor workers compared with indoor workers [42]. This pattern of incidence among workers has usually been attributed to the fact that a more continuous pattern of sun exposure may reduce the incidence of sunburns, a known melanoma risk factor. However, an alternative hypothesis is that outdoor workers are less likely to be deficient in vitamin D because of their more regular exposure to sunlight.

Seasonal variation in serum 25(OH)D levels is well known [43], and the winter level of serum 25(OH)D is considered the best indicator of an individual’s real vitamin D status.

Boniol *et al* showed significant variations in incidence and fatality of melanoma with relation to season of diagnosis. There was also a 16% (95% CI: −6%; −28%) decrease in the multivariate adjusted fatality rate between winter and summer after adjustment for Breslow thickness, suggesting other factors apart from earlier diagnosis influence survival, for instance a late promotional effect of greater exposure to UV in the summer [44]. An Italian study found significantly increased survival in melanoma patients who had intermittent sun exposure before diagnosis [45].

A study population of 212 patients with histologically proven cutaneous melanomas showed that progression of malignant melanoma was associated with statistically significantly reduced 25(OH)D serum levels [46].

### Vitamin D and melanoma, biological evidence

Cultured melanoma cells can synthesize 1,25(OH)_2_D_3_ from 25(OH)D_3_, express the VDR and proliferate more slowly in response to 1,25(OH)_2_D_3_ [47–49]. Moreover, 1,25(OH)_2_D_3_ has been shown to suppress the growth of human malignant melanoma (MM)-derived xenografts (expressing the VDR) in immunosuppressed mice, but not in a VDR-negative MM cell line. The 1,25(OH)_2_D_3_ molecule has been shown to induce apoptosis in a human MM cell line *in vitro*, and an inhibitory effect on the spread of MM cells has been demonstrated *in vitro* [50].

Upon the activation by 1,25(OH)_2_D_3_, the VDR mediates its effects by regulating the transcription of other genes. The VDR gene is located on chromosome 12q12–q14, and it has at least 196 single nucleotide polymorphisms, some of which seem to influence the activity of 1,25(OH)_2_D_3_. Among VDR polymorphisms, the most frequently investigated for their association with various cancers are FokI (exon 2, rs10735810), which results in an altered translation start site and has been shown to be functionally relevant [51], and BsmI (intron 8, rs1544410), which seems to be associated with different diseases, although its function is still under debate [52,53].

A meta-analysis on vitamin D receptor polymorphisms and skin cancer

A meta-analysis suggests a possible significant role of VDR ***Fok***I and ***Bsm***I polymorphisms in CMM and non-melanoma skin cancer (NMSC): the ***Fok***I f allele showed a positive association with CMM and NMSC, whereas the ***Bsm***I B allele showed a significantly negative association with CMM [54]. Further studies on the role of VDR polymorphisms in skin cancer development could be useful to better understand these associations.

### Serum levels of 25(OH)D, VD R and melanoma prognosis

A cohort study was carried out in Leeds (UK) to test the hypothesis that higher vitamin D levels reduce the risk of relapse from melanoma. A prospective survival analysis in this cohort of 872 patients showed that higher 25-hydroxyvitamin D3 levels, at diagnosis were associated with lower Breslow thickness at diagnosis (p=0.002) and were independently protective of relapse and death: the hazard ratio for relapse-free survival (RFS) was 0.79 (95% CI, 0.64 to 0.96; p=0.01) for a 20 nmol/l increase in serum level. There was evidence of interaction between the vitamin D receptor (VDR) BsmI genotype and serum 25-hydroxyvitamin D3 levels on RFS [55].

Two studies evaluating serum levels of 25(OH)D and skin cancer risk [46,56] reported lower basal 25(OH)D levels in melanoma patients compared to the control group, although this difference was statistically not significant. Bishop evaluated the risk of relapse for CMM patients taking vitamin D supplementation and found a significant reduced risk: 0.54 (0.32–0.92). The results were presented as an abstract publication [56]. Moreover, Nurnberg [46] found that progression of malignant melanoma was associated with statistically significantly reduced 25(OH)D serum levels. Two other studies found an association of ***VDR*** polymorphisms with Breslow thickness [57,58], suggesting a role for vitamin D in the CMM prognosis.

Halsall *et al* evaluated also a novel adenine-guanine substitution −1012 bp relative to the exon 1a transcription start site (A-1012G) of the vitamin D receptor. The ***A*** allele was over-represented in CMM patients (p=0.007). The outcome was related to the development of metastasis, the Kaplan-Meier estimates of the probability of metastasis at five years being: ***GG*** 0%; ***AG*** 9%, CI 4–16%; ***AA*** 21%, CI 12–36%; (p=0.008), and to thicker Breslow thickness groups (p=0.04). The effect on metastasis was independent of tumour thickness and A-1012G may have predictive potential, additional to Breslow thickness. ***Fok***I and ***Taq***I variants were strongly related to the thickest Breslow thickness group (p=0.005). There was an interaction between the A-1012G and ***Fok***I polymorphisms (p=0.025) and the ***Fok***I variant enhanced the effect of the ***A*** allele of the ***A***-1012G polymorphism on metastasis, the probability of metastasis for ***AAff*** at a five-year follow-up being 57%, CI 24–92% [57].

### The need for a trial on vitamin D supplementation

Vitamin D is a drug, more precisely, a hormone. The experience accumulated in the last 20 years with chemoprevention and hormonal substances shows that no compound should be recommended for cancer chemoprevention if its efficacy and side effects have not been evaluated in large, randomized trials. Laboratory data and observational studies should only be considered as indicative of potential for chemopreventive use.

Findings from prospective cohort studies on colorectal cancer risk and on mortality constitute pieces of evidence strong enough to consider that randomized trials of vitamin D use and cancer mortality may not have correctly addressed the question, and that new randomized trials should be organized.

Some groups advocate increasing vitamin D status (e.g. above 30 ng/ml of serum 25-hydroxyvitamin D) through more exposure to ultraviolet radiation or through taking high doses of vitamin D supplements (i.e. more than 50 μg/d or more). However, sun exposure is also a well-known risk factor for certain cancers and, as for any drug, before issuing claims on health benefit and promoting recommendations for increasing substantially the vitamin D status of millions of individuals, the alleged claims must be tested via randomized controlled trials for evaluation of efficacy on primary endpoint(s) [59].

### The issue of the dose

The estimated optimal serum 25(OH)D for prevention of cancer equal to or greater than 30 ng/ml is a conclusion supported by the NIH-sponsored symposium on vitamin D and cancer [18,19].

Results from the meta-analyses on 25(OH)D serum levels and cancer incidence, within the working group of experts, organized by the International Agency for Cancer Research, showed a significant reduction in risk for colorectal cancer comparing the highest levels versus the lowest level of 25(OH)D, with a significant dose-response effect [59]. Among the studies included, the lowest values of 25(OH)D for the upper categories on average were about 30 mg/ml and the upper levels of the lowest category was 18 mg/ml.

In a trial (IEO 007) carried out by the Cancer Prevention and Genetics Division of the European Institute of Oncology in pre-menopausal women at high risk of breast cancer, we found that 85% had insufficient levels of 25(OH)D, considering 30 ng/m as cut-off point.

It has been calculated that 2000 IU/d should shift the NHANES III distribution so that only 10–15% of persons had concentrations <30 ng/ml, which are observed in healthy outdoor workers.

This dose is not toxic and necessary to achieve the optimal level of 25(OH)D. A daily dose of 2000 IU vitamin D_3_, the safe upper intake limit as defined by the National Academy of Sciences, should achieve the desired level of circulating 25(OH)D [17,19,24,26].

Cholecalciferol is accumulated in adipose and muscular tissue to be available when the body needs it. Vitamin D intoxication is extremely rare but can be caused by inadvertent or intentional ingestion of excessively high doses. Doses of more than 50,000 IU/d raise levels of 25-hydroxyvitamin D to more than 150 ng/ml (374 nmol/l) and are associated with hypercalcemia and hyperphosphatemia [30,31,60]. All of the reports of vitamin D toxicity showing the convincing evidence of hypercalcemia involve serum 25(OH)D concentrations well above 200 nmol/l, which requires a daily intake of more than (40,000 IU), and which could be conservatively considered the lowest observed adverse effect level [28,29]. The Women’s Health Initiative trial reported a 17% increase in the risk of kidney stones among post-menopausal women receiving 10 μg of vitamin and 1000 mg of elemental calcium per day ^34^, but this increase was probably due to the calcium supplements, and not to the vitamin D. In fact other randomized trials testing vitamin D supplementation did not report higher incidence of kidney stones [4].

As a result of these studies, we decided on 100,000 IU every 50 days, which corresponds to 2000 IU daily, for the following trial on melanoma patients.

## Summary of background and specific aims of an Italian multi-centre randomized placebo-controlled phase III trial

Vitamin D and cancer: calcitriol, the biologically active form of vitamin D (1,25(OH)D), exerts its effects mainly through binding to the nuclear VDR. Calcitriol has been shown to be an anti-proliferative, pro-differentiation, pro-apoptotic agent and an inhibitor of cell migration. Animal and human *in vitro* studies strongly indicate that vitamin D may be beneficial for many forms of cancer. Inadequate levels of circulating 25-hydroxy-vitamin D (25(OH)D) are associated with an increased risk and poor prognosis of several types of cancer.

Vitamin D and melanoma: cutaneous malignant melanoma represents a major public health problem—rates in Italy have almost doubled in the last decade and CMM is frequent among young adults. For resected stage II melanoma no standard adjuvant treatment exists and five-year overall survival is about 70%.

Cultured melanoma cells can synthesize calcitriol from 25(OH)D and express the VDR. Moreover, the anti-proliferative and pro-differentiation effect of 1,25(OH)D have been found in human melanoma cells. 1,25(OH)D has been shown to induce apoptosis in human melanoma cell lines and an inhibitory effect on the spread of melanoma cells has been demonstrated *in vitro*.

Preliminary results on vitamin D: Epidemiological data indicate that vitamin D deficiency is relatively common in Europe. In an Italian study, we found that 85% of the participants had insufficient level of 25(OH)D. We show through a meta-analysis of randomized trials that vitamin D supplementation was associated with a significant reduction of 7% in total mortality in healthy subjects. We also reported significant associations between VDR polymorphisms and incidence of skin cancer while other studies suggest association between VDR and 25(OH)D and CMM progression. In early supplementation trials, the lack of effect on cancer incidence has been attributed to insufficient vitamin D supplementation, stressing the need to better study vitamin D bioavailability.

Finally, a recent IARC report highlighted the need for new randomized trials, based on results from our meta-analyses on 25(OH)D serum levels and cancer risk.

## Conclusions

Several epidemiological, pre-clinical and clinical studies support vitamin D as a preventive and therapeutic cancer agent, for a wide spectrum of cancer. The aim of this proposed study is to evaluate the effect of vitamin D supplementation on melanoma recurrence and mortality.

## Overview of study design

We propose a randomized double-blind placebo-controlled clinical trial to evaluate whether vitamin D supplementation and changes of serum levels of 25(OH)D are associated with melanoma recurrence and mortality. Patients with resected stage II melanoma will be recruited in different institutes in Italy. Participants will be randomly assigned to one of the arms (100,000 IU po every 50 days, an average 2000 IU a day, versus placebo), treated for three years and followed for two years.

Recruitment will proceed simultaneously at the different centres and will be completed in four years. Complete physical examination, measurements of anthropometric and epidemiological characteristics, as measure of study compliance will be performed at baseline as well as at the interim visits, every four months. We will collect blood samples to measure vitamin D levels every four months for the first year, and annually thereafter. Vitamin D polymorphisms will be evaluated at baseline. Efficacy will be determined by assessing CMM recurrence as the primary outcome. Three are the planned interim analyses. We will also evaluate general and CMM specific mortality.

Compliance will be evaluated by self-reported number of ampoules taken, and supported by serum 25(OH)D_3_ levels.

The study will be conducted across Italian Institutes sponsored by the European Institute of Oncology. The minimum length of the follow-up period will be two years, and the end of follow-up is determined by recurrence, death or last day of the follow-up.

## Aims of the trial

### Primary aim

To assess the effect of Vitamin D_3_ supplementation (2000 IU/day) on disease-free survival for stage II CMM patients through a randomized, placebo-controlled trial.

### Secondary aims

To evaluate:

- whether vitamin D receptors (***Bsm***I, ***Fok***I and A-1012G polymorphisms), gene–gene interactions and serum level of 25(OH)D are associated with melanoma prognosis: we will assess the association at baseline between 25(OH)D serum levels, vitamin D receptor (VDR) polymorphisms and gene–gene interactions with Breslow thickness, the main prognostic factors;
- whether changes in 25(OH)D are associated with DFS or OS;
- whether Vitamin D receptors, CYP27A1, CYP24A1 and GC (vitamin D binding protein) genes are associated to serum level of 25(OH)D at baseline and with changes in 25(OH)D levels;
- whether 25(OH)D serum levels of vitamin D are associated with BMI, vitamin D food intake and fat intake;
- whether vitamin D receptors and 25(OH)D serum levels of vitamin D are associated with other prognostic factors such as number of mitosis, gender, age, tumour location and ulceration;
- percentage of patients at 30 mg/ml level of 25(OH)D at one year and mean time to reach that level;
- change in overall survival (OS) in melanoma patients at stage II, taking vitamin D supplementation;
- the pattern of response of parathormone (PTH) following vitamin D_3_ supplementation in these patients, and association with changes in 25(OH)D;
- 25(OH)D serum levels and anthropometric measures (such as BMI, waist-to-hip ratio, waist-to-stature ratio), dietary intakes (vitamin D, calcium and fat intake), occupation and different patterns of sun exposures;
- Short and long-term safety and toxicity at 2000 IU/d;
- Compliance.

Finally, we will develop a bio-repository of blood samples, collected at several time points. This will create a large biological database for future research (e.g. molecular characteristics of intervention efficacy, serum, vitamin D serum levels).
